# Long-Term Survival of a Patient with Brainstem and Recurrent Brain Metastasis from Stage IV Nonsmall Cell Lung Cancer Treated with Multiple Gamma Knife Radiosurgeries and Craniotomies: A Case Report and Review of the Literature

**DOI:** 10.1155/2012/621641

**Published:** 2012-09-29

**Authors:** Andrew F. Lamm, Ameer L. Elaimy, Alexander R. Mackay, Robert K. Fairbanks, John J. Demakas, Barton S. Cooke, Christopher M. Lee, Blake S. Taylor, Wayne T. Lamoreaux

**Affiliations:** ^1^Gamma Knife of Spokane, Deaconess Health and Education Building, 910 W 5th Avenue, Suite 102, Spokane, WA 99204, USA; ^2^Cancer Care Northwest, Deaconess Health and Education Building, 910 W 5th Avenue, Suite 102, Spokane, WA 99204, USA; ^3^MacKay & Meyer MDs, Spokane, WA 99202, USA; ^4^Spokane Brain & Spine, Spokane, WA 99204, USA

## Abstract

The prognosis of patients diagnosed with stage IV nonsmall cell lung cancer that have brain and brainstem metastasis is very poor, with less than a third surviving a year past their initial date of diagnosis. We present the rare case of a 57-year-old man who is a long-term survivor of brainstem and recurrent brain metastasis, after aggressive treatment. He is now five and a half years out from diagnosis and continues to live a highly functional life without evidence of disease. Four separate Gamma Knife stereotactic radiosurgeries in conjunction with two craniotomies were utilized since his initial diagnosis to treat recurrent brain metastasis while chemoradiation therapy and thoracic surgery were used to treat his primary disease in the right upper lung. In his situation, Gamma Knife radiosurgery proved to be a valuable, safe, and effective tool for the treatment of multiply recurrent brain metastases within critical normal structures.

## 1. Introduction

Brainstem metastases are a particularly difficult oncological and neurological clinical problem. In most cases, these lesions are inoperable and carry a grim prognosis [1, 2]. Furthermore, most chemotherapeutic agents cannot pass through the blood brain barrier making chemotherapy ineffective. Whole-brain radiation therapy and stereotactic radiosurgery (SRS) are the most commonly used techniques for the treatment of brainstem metastasis. Radiation therapy rests on the principal that the unregulated cell cycles of cancerous cells in the brain have a faster turn over rate and a reduced ability to repair DNA damage. Whole-brain radiation allows for safe treatment over large areas of the brain at a low daily dose to preferentially damage cancer cells. Stereotactic radiosurgery is considered an alternative to neurosurgery or whole-brain radiotherapy for select cases. SRS uses a high dose of radiation to specifically target cancerous lesions within the brain. SRS can also be used as a boost after conventionally fractionated radiotherapy.

 This paper presents the case of a man who underwent aggressive treatment for stage IV metastatic lung cancer with a subsequent pontine brainstem metastasis and other recurrent brain metastases. He underwent four separate stereotactic radiosurgeries with a Leskell Gamma Knife and two craniotomies for treatment of his brain lesions. He also received chemotherapy, radiation therapy, and thoracic surgeries to treat the primary lung cancer. Despite his challenging situation, he is currently a long-term survivor and is now five and a half years out from his stage IV diagnosis, with no current evidence of disease.

## 2. Case Presentation

 In December of 2006, a 57-year-old man presented with severe right upper posterior chest and back pain. A chest X-ray revealed a mass in the right upper chest. A subsequent CT scan confirmed an 8 cm mass in the posterolateral right upper lobe (RUL) with destruction of the adjacent ribs and invasion into the chest wall. Moreover, there was a separate spiculated mass in the contralateral left upper lobe (LUL) measuring 11 mm. A PET/CT showed the large RUL mass with a standardized uptake value (SUV) of 14, and the LUL lesion had an SUV of 3.4. There was no significant hypermetabolism in the mediastinum and there was no evidence of distant disease.

**Figure 1:**
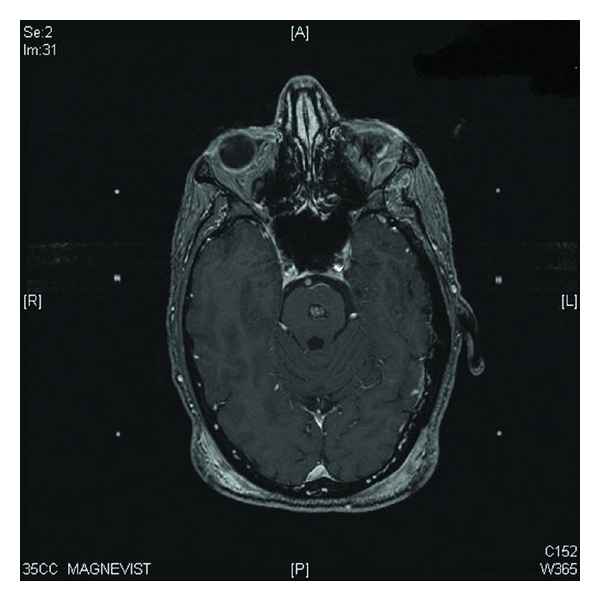
Axial T1 postgadolinium enhanced brain MRI with a visible pontine brain metastasis.

**Figure 2:**
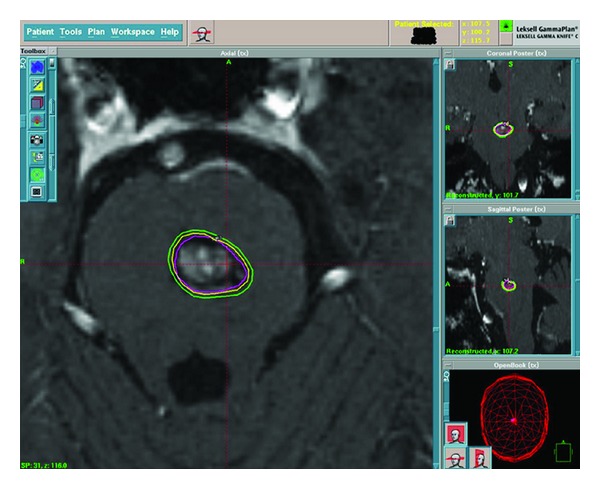
Axial T1 postgadolinium enhanced treatment planning brain MRI illustrating the targeting ability and radiation isodose lines of Gamma Knife radiosurgery for the pontine metastasis.

In January 2007, he underwent a cervical mediastinoscopy and left video-assisted thoracoscopy with wedge resection of the left upper lobe lesion. The LUL pathology showed a moderately to poorly differentiated adenocarcinoma measuring 1.0 cm. All of the mediastinal lymph nodes were negative. It was unclear whether or not the LUL nodule was a separate primary or a metastatic lesion from the RUL primary mass. A brain MRI showed a 6 × 6 mm lesion in the midline pons with minimal hemorrhage and mild surrounding edema that was strongly consistent with a single metastatic lesion (see Figure 1). Therefore staging was determined to be T3N0M1, Stage IV, and it was felt that his primary disease should be treated aggressively secondary to chest wall invasion and pain and limited metastatic disease. He was started on chemoradiotherapy to the RUL primary, and Gamma Knife radiosurgery was performed the next month to target the lesion in the pons. A prescription dose of 14 Gy to the 50% isodose line was used, which resulted in 100% of the tumor receiving 15.1 Gy (see Figure 2 for an example of the treatment planning process). The conformality index was excellent at 1.2.

**Figure 3:**
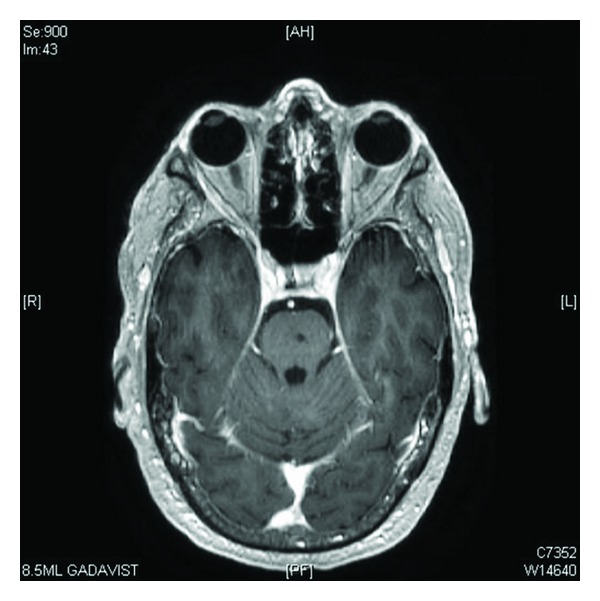
Posttreatment axial MRI image of the pons with complete ablation of the pontine metastasis and no further visible disease within the brainstem.

He completed 60 Gy in 30 fractions via a 3D conformal radiotherapy course with concurrent carboplatin and Taxol to the RUL and chest wall mass. In April 2007, a follow-up brain MRI showed an excellent response to the GKSRS with no residual enhancement in the pontine metastasis and no new brain lesions (see Figure 3). In May of 2007, a restaging PET/CT showed 50% reduction in the size of the RUL tumor with reduced SUV of 8, but still with significant invasion of the chest wall, yet still no evidence of distant disease. Therefore, with excellent performance status, the patient elected to undergo a right thoracotomy, right upper lobectomy, and extensive chest wall resection. The primary tumor was excised, with good margins and without complication. The tumor-infiltrated region of the chest wall was resected along with large portions of the third and fourth ribs. Pathology revealed 99% necrotic poorly differentiated nonsmall cell carcinoma with few viable cells left in the specimen. 

A follow-up MRI two months later illustrated that the lesion in the mid pons was no longer visible as an enhancing lesion and that the signal intensity of the lesion had decreased significantly. However, there were two new brain metastases. One 5 mm lesion in the right cerebellum was identified, and another 6 mm lesion was seen in the frontal corticomedullary junction.

The patient desired to avoid whole-brain radiation and elected to proceed with a second Gamma Knife treatment in July 2007. The two lesions were targeted with a prescription dose of 20 Gy to the 50% isodose line. It was noted that the lesions had grown in the interim with the right cerebellar lesion now 13 mm and the frontal lesion was 14.2 mm, respectively.

An interim MRI showed good response; however an MRI in January 2008 demonstrated that the right superior frontal metastasis had increased significantly in size to 27 × 27 mm and was accompanied with increased vasogenic edema. This was consistent with either progression or pseudoprogression (radiation change). The patient elected to proceed with a stealth stereotactic craniotomy with microsurgical tumor resection. A follow-up CT of the head showed that the craniotomy removed most of the enhancing large lesion; nevertheless, some residual enhancement along the posterior rim remained. 

During a followup three months later, an MRI illustrated a new enhancing focus adjacent to the anterior margin of the resected lesion and the interval increase in the surrounding vasogenic edema compatible with disease progression. The patient underwent his third Gamma Knife radiosurgery in May 2008, with a prescription dose of 18 Gy to the 50% isodose line. The follow-up MRI showed a modest response in the treated right frontal lesion. An MRI two months later showed progression of the metastatic disease in the right frontal lobe with an increased interval of nodular peripheral margin enhancement to 2.7 × 2.2 × 2.7 cm, with surrounding reactive edema. He maintained good performance status and elected to proceed with a reoperative craniotomy later in Oct 2008. The tumor bed was excised successfully, and pathology confirmed 90% necrotic metastatic carcinoma consistent with a lung primary. There was no active disease at the margins. The follow-up MRI confirmed the absence of residual enhancement. 

The patient was still adamantly against whole brain radiation or even a more limited fractionated radiotherapy course. Due to the extensively resilient nature of his local brain disease in the right frontal lobe, the patient opted to complete a final Gamma Knife radiosurgery to the resection margin in November 2008. The previously treated cerebellar tumor was seen as a tiny enhancing lesion on the planning MRI scan, which was not seen on previous scans. It was treated with 9 Gy to the 50% isodose line (16.8 Gy marginal dose). The right frontal resection bed was treated to 15 Gy to the 50% isodose line. 

Subsequent MRIs have shown steady response and no definite evidence of disease. Currently he has no evidence of systemic or intracranial disease. He tolerated all the treatments remarkably well.

He denies any significant problems, although he states that he has some difficulty with memory problems. He has no neurologic problems and no headaches. The patient has made a remarkable recovery and has toiled his way through a potentially fatal prognosis to become a long-term survivor. He is leading a normal active life at this point; in fact, he recently completed construction on his own new home.

## 3. Discussion

The prognosis for patients with brainstem metastasis is very poor with a median survival times ranging from 4 to 12 months [1, 3–5]. The chance for long-term survival at one year is approximately 30% according to a study done by Hussain et al. [6]. Our patient has had an incredible recovery even though he carried a diagnosis wherein most patients have an extremely poor prognosis. 

 There are several patient selection factors that advocate for the use of stereotactic radiosurgery. These include lower tumor radiosensitivity, smaller tumor size, spatial positioning of tumor in or near a delicate area of the brain (i.e., the brainstem, optic chiasm), and a lower overall number of metastasis. In a previous review written by Hazard et al. [7], other factors that suggest patients who would benefit from stereotactic radiosurgery are good performance status, controlled primary, and systemic disease. 

 Furthermore, a review of the literature reveals a current controversy over which treatment modality, or combination thereof, provides the best treatment and survival probability for the patient. In a report written by Aoyama et al. [8], the researchers compared patients diagnosed with 1–4 brain metastasis treated with stereotactic radiosurgery to patients treated with stereotactic radiosurgery plus whole-brain radiation therapy. They found that the survival rates of patients who were treated with stereotactic radiosurgery alone did not differ significantly when compared to those who were treated with both stereotactic radiosurgery and whole-brain radiation therapy. However, patients treated with both modalities relapsed less often and required fewer salvage treatments. A report by Andrews et al. [9] noted that when patients treated by whole-brain radiation therapy alone were compared to patients treated with both SRS and whole-brain, the patients who received both treatments demonstrated longer survival times. However, a trial by Patchell et al. [10] found that the addition of whole-brain radiosurgery to surgery did not have an impact on survival but decreased recurrence from 70% to 18%. 

Gamma Knife has been used for decades and is safe, but there are possible side effects that might result from treatment. In a study by Sharma et al. [11], the authors address the radiation limits of the brainstem and offer insight about the chances of suffering a neurological defect as a result of radiation treatment. They found that four patients from their study group of thirty-eight patients developed transient neurological defects including facial numbness, paresthesia, ataxia, and nystagmus. The researchers also suggest a dose of 12 Gy as a safe limit to the brainstem. However, they acknowledge that 15% of their patient cohort received greater than 18 Gy with only a solitary case of neurological deficit. This suggests that while caution must be exercised for treatments near the brainstem, a higher marginal dose may still be safe. Another article by Shuto et al. [12] concluded that a marginal dose of 15 Gy to patients with brainstem metastasis is effective and relatively safe. They found that in a patient cohort of twenty-five, only two patients developed radiation-induced injury. The mean prescription dose was 13 Gy. Other possible side effects of Gamma Knife radiosurgery include headaches, seizures, nausea, edema, loss of balance, and vision problems [13]. The literature suggests that the brainstem, while delicate, may be a more resilient structure than was initially thought. Gamma Knife radiosurgery to the brainstem is both safe and effective as a treatment for brainstem metastasis. 

 Against odds, our patient so far has been successfully treated for stage IV lung cancer and brain metastasis. Gamma Knife radiosurgery in conjunction with craniotomy can be used effectively in the aggressive treatment of brainstem metastasis with recurrent brain metastasis. The precision of stereotactic radiosurgery to treat cancer-infiltrated regions near delicate areas without causing undue damage to the surrounding tissues makes it a safe and ideal treatment. The patient is now cancer-free and continues to live a healthy high quality life.
